# Fabrication of Membrane Sensitive Electrodes for the Validated Electrochemical Quantification of Anti-Osteoporotic Drug Residues in Pharmaceutical Industrial Wastewater

**DOI:** 10.3390/molecules26165093

**Published:** 2021-08-22

**Authors:** Sherif A. Abdel-Gawad, Obaid Afzal, Hany H. Arab, Alhumaidi B. Alabbas, Abdulmalik M. Alqarni

**Affiliations:** 1 Department of Pharmaceutical Chemistry, College of Pharmacy, Prince Sattam Bin Abdulaziz University, Al Kharj 11942, Saudi Arabia; obaid263@gmail.com (O.A.); ab.alabbas@psau.edu.sa (A.B.A.); 2 Analytical Chemistry Department, Faculty of Pharmacy, Cairo University, Cairo ET-11562, Egypt; 3 Department of Pharmacology and Toxicology, College of Pharmacy, Taif University, P.O. Box 11099, Taif 21944, Saudi Arabia; h.arab@tu.edu.sa; 4 Department of Pharmaceutical Chemistry, College of Clinical Pharmacy, Imam Abdulrahman Bin Faisal University, Dammam 31441, Saudi Arabia; amalqarni@iau.edu.sa

**Keywords:** electrochemistry, glassy carbon electrode, ion selective electrode, PVC-based membrane electrode, wastewater

## Abstract

Accurate and precise application of ion-selective electrodes (ISEs) in the quantification of environmental pollutants is a strenuous task. In this work, the electrochemical response of alendronate sodium trihydrate (ALN) was evaluated by the fabrication of two sensitive and delicate membrane electrodes, viz. polyvinyl chloride (PVC) and glassy carbon (GC) electrodes. A linear response was obtained at concentrations from 1 × 10^−5^ to 1 × 10^−2^ M for both electrodes. A Nernstian slope of 29 mV/decade over a pH range of 8–11 for the PVC and GC membrane electrodes was obtained. All assay settings were carefully adjusted to obtain the best electrochemical response. The proposed technique was effectively applied for the quantification of ALN in pure form and wastewater samples, acquired from manufacturing industries. The proposed electrodes were effectively used for the determination of ALN in real wastewater samples without any prior treatment. The current findings guarantee the applicability of the fabricated ISEs for the environmental monitoring of ALN.

## 1. Introduction

Bisphosphonates are the therapeutic agents used to treat postmenopausal bone loss, Paget’s disease, malignancy-related hypercalcemia, and osteolytic osseous metastatic disease [1]. The common use of these drugs may lead to their presence in the environment and can cause serious effects on human health. Bisphosphonates are not metabolized and excreted unchanged in the urine, which leads to their accumulation in hospital sewage. They are generally found in surface and underground water, due to their polar and water-soluble nature. In addition, their presence in pharmaceutical wastewater may constitute a great risk to human health. Long-term intake of bisphosphonates may lead to many adverse effects including acute tubular necrosis, nephrotoxicity, and jaw osteonecrosis [1,2]. Regarding numerous studies on the determination of pharmaceutical agents in wastewaters and their hazardous effects on the health of living beings, there is no fixed limit for their presence in soil or natural water [3].

**Figure 1:**
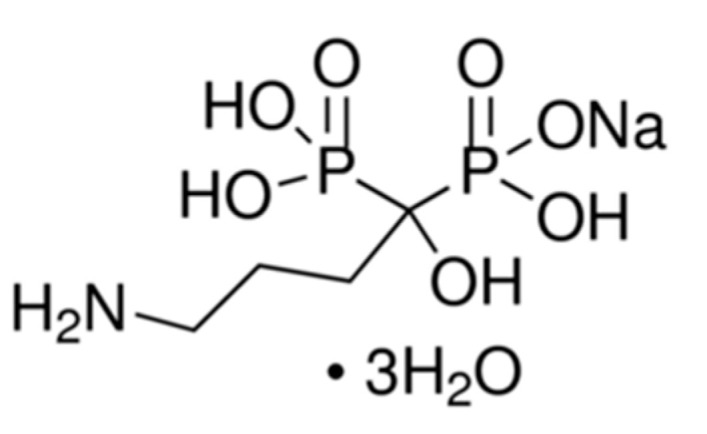
Chemical structure of alendronate sodium trihydrate (ALN).

Alendronate sodium trihydrate (ALN) is the most commonly used bisphosphonate, so it is included in this study to monitor its level in pharmaceutical wastewater. ALN (IUPAC name: sodium trihydrate hydrogen (4-amino-1-hydroxy-1-phosphonobutyl)phosphonate) (Figure 1) belongs to the category of bisphosphonates used for the treatment of Paget’s disease and other osteolytic diseases [4].

Regarding the reported analytical methods used to detect and quantify ALN, spectrophotometric methods were developed to determine ALN, either by complexation with ferric ions [5] or by the formation of charge-transfer complexes with π-electron acceptors like 7,7,7,8-tetracyanoquinodimethane (TCNQ) and 2,3-dichloro-5,6-dicyano-1,4-benzoquinone (DDQ) [6]. Additionally, it was determined spectrophotometrically via the reaction of its primary amino group with some reagents like ninhydrin [7] and 4-chloro-7-nitrobenzo-2-oxa-1,3-diazole (NBD-Cl) [8]. RP-HPLC columns are not suitable for the retention of ALN, due to their strongly polar and ionic nature [9]. Additionally, due to its non-volatile nature and absence of strong chromophores for sensitive UV and fluorescence detection, it is hard to quantify by standard analytical techniques like HPLC or gas chromatography (GC). However, some ion pair and ion exchange chromatographic methods have been developed for the direct quantification of ALN, but those methods are not suitable for its detection in wastewaters [10,11,12,13].

Some HPLC (ultraviolet or fluorescence detection) [14,15,16,17] and LC-MS/MS [18,19,20] methods were also reported that involve derivatization techniques for the quantification of ALN. The derivatization technique aims to introduce a chromophore or fluorescent label to ALN to increase its analytical sensitivity. These methods are labor-intensive, complicated, and suffer from drawbacks like low selectivity. On the other hand, most of the methods applying the LC-MS/MS technique are dependent on derivatization using amino-containing reagents, like diazomethane. It is an unstable and unsafe reagent that requires special safety precautions. Additionally, the LC-MS/MS technique is a complicated multi-step, non-economic technique [21]. Moreover, sample preparation and extraction processes are also required to remove the matrix effect. Therefore, there is an urgent need to develop an economic and simple method that can bypass the tedious and complicated procedures used in sample pre-treatment during ALN quantification.

Electrochemical sensor systems have the capability to quantitatively analyze particular chemical species in short response times with sufficient precision, selectivity, and sensitivity [22,23]. Therefore, we focused on developing and validating an easy-to-use and economical method to screen and quantify ALN in wastewater samples by using ion-selective electrodes (ISEs) as an analytical means for environmental monitoring and analysis. ISEs are commonly used for the determination of organic and inorganic compounds for their frequent analysis, due to being less time consuming, low cost, and their non-destructive approach [24]. It is obvious from the literature review that there is no published work dealing with the electrochemical quantification of ALN in environmental wastewater samples. Therefore, the aim of this work is the development and application of ISEs for straightforward, simple, careful, and profound quantification of ALN in diverse samples, particularly in industrial wastewater and complex matrices, without any prior treatments.

## 2. Results

**Figure 2:**
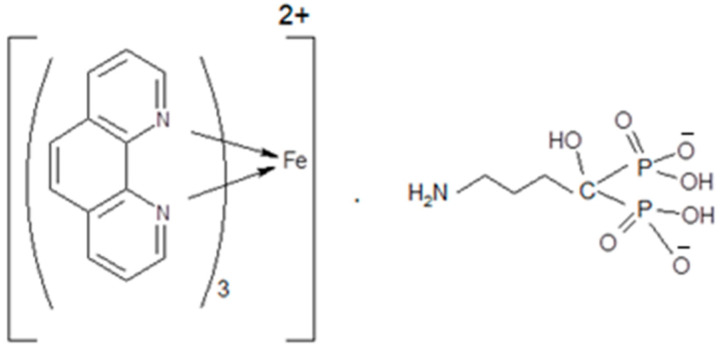
Structural formula of ALN *o*-phenanthroline-Iron (II) association complex.

This work demonstrates the capability of selective quantification of ALN through the fabrication of sensitive membrane sensors. The fabricated sensors are used to quantify ALN residues in wastewater samples without sample pre-treatment, which in turn, makes the environmental monitoring task of the studied drug an easy process. ALN acts as a bivalent ion with the ion exchanger, and forms a stable 1:1 ion association complex with *o*-phenanthroline-Iron (II), as evidenced by the elemental analysis of the formed complex (Table 1). The formed complex structural formula is illustrated in Figure 2.

**Scheme 1:**
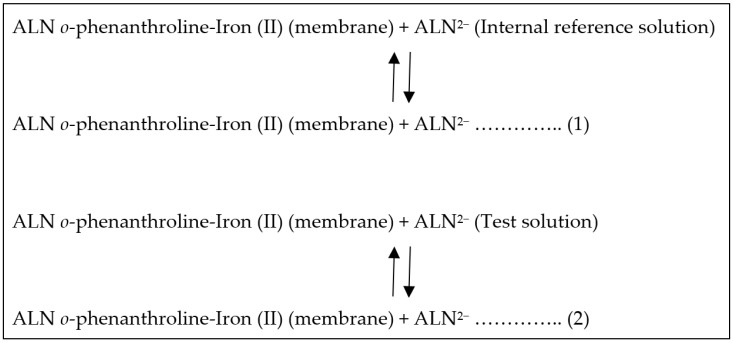
Schematic representation for the reaction mechanism showing ALN exchange across the fabricated membrane.

The three main components of taking a measurement with an ISE are an internal reference or standard solution and an outer analyte, or sample solution separated by a thin membrane. The potential developed at the membrane is the result of either an ion exchange process or an ion transport process occurring at each interface between the membrane and solution. At each solution–membrane interface, an ion exchange equilibrium is established (Scheme 1). The partitioning of ALN between the aqueous solution phase and the membrane phase depends on its activity or concentration. The resulting charge separation at each interface results in a phase-boundary potential [25,26,27].

### 2.1. Performance Characteristics of PVC Membrane Electrode and GCE

**Figure 3:**
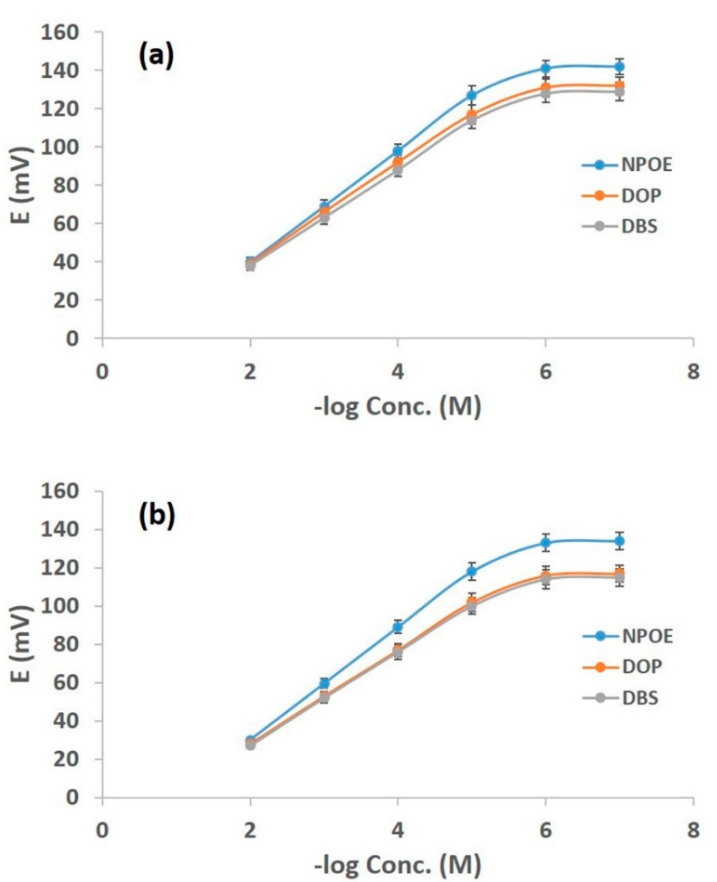
Profile of the potential (in mV) versus –log concentration (in M) for (**a**) PVC membrane electrode and (**b**) GC electrode at pH 9, using different solvent mediators.

The execution of ISEs was assessed by various factors including response time, pH range, dynamic linear response ranges, the limit of detection, selectivity, and shelf life. The electrochemical performance features of the ISEs were examined as per the IUPAC standards [28]. The effect of solvent mediators was studied using three different plasticizers of different polarities. They were dioctyl phthalate (DOP), nitrophenyl octyl ether (NPOE), and dibutyl sebacate (DBS). NPOE as a plasticizer produced the perfect response as the slope of its fabricated sensors was closest to the Nernstian slope. The calibration slope for the PVC and GC membrane electrodes was found to be 29 mV/decade, for both electrodes (Table 2). The slight deviation from the model Nernstian slope (30 mV/decade) may be attributed to the fact that the electrode responds to the activities of the drug rather than its concentration. Typical calibration curves are depicted in Figure 3.

**Figure 4:**
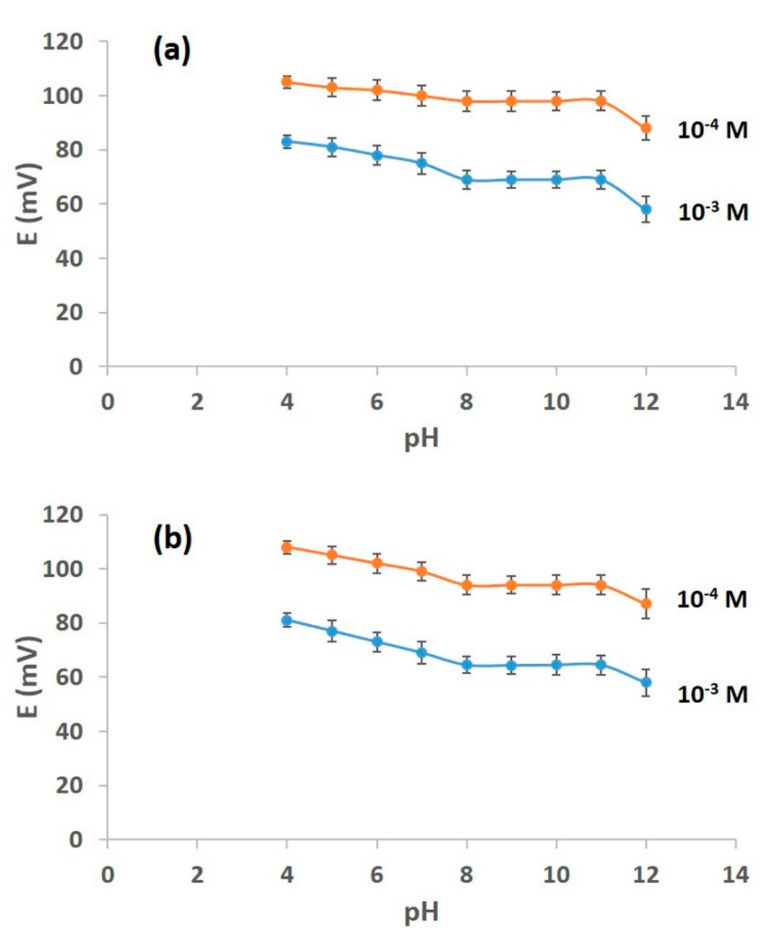
Effect of pH on the potential response of (**a**) PVC membrane electrode and (**b**) GC electrode.

The linearity range was 1 × 10^−5^ to 1 × 10^−2^ M for the target drug ALN, in a PVC membrane electrode and GC electrode. The PVC membrane electrode displayed almost constant potential readings (±2 mV/decade) for almost 25 days. On the other hand, the GC electrode displayed a lifetime of 30 days. The sensors displayed a fast and steady response with a low limit of detection (LOD) over a wide range of concentrations (Table 2). The pH effect on the electrochemical response of the electrodes was examined at a pH range of 4 to 12, using concentrations of 1 × 10^−3^ M and 1 × 10^−4^ M of ALN (Figure 4). A constant response was attained in the pH ranges of 8 to 11 for the PVC and GC membrane electrodes. The response was noisy and exhibited considerable aberrations from the model Nernstian slope (30 mV/decade) besides this pH range.

The calibration plots were also studied at three temperature levels of 25 °C, 30 °C, and 40 °C, to study the effect of temperature on the electrodes’ potential. It was observed that the constructed calibration graphs were almost similar. The LOD and slope showed insignificant variation, indicating realistic thermal stability of the fabricated membrane electrodes up to 40 °C. The results regarding the thermal stability of electrodes at three temperature levels are tabulated in Table 3.

The accuracy of the cited method was checked by analyzing pure ALN samples of different concentrations. Additionally, the ruggedness and robustness of the suggested analytical method were carefully ensured, as pointed out in Table 2. Selectivity of the fabricated membranes is an important parameter in performance optimization, as it defines the scope and usefulness of any sensor in real sample measurements. The response of fabricated membranes for the sample analyte in the presence of interfering materials was analyzed in terms of the potentiometric selectivity coefficient (PSC) [29]. The PSC defines the preference for the sample analyte by the membranes as compared to an interfering material. It has an advantage, as it bypasses the limitations imposed by the Nicolsky–Eisenman equation. Those limitations comprise non-Nernstian conduct of interfering ions and dissimilar charges of studied and interfering ions. Different salts and related bisphosphonate drugs were tried as interfering substances to check the selectivity of the fabricated electrodes. The calculated PSCs are shown in Table 4. The PSCs were of the order of 10^−2^ or even smaller, showing excellent selectivity of fabricated sensors.

### 2.2. Determination of ALN in Spiked Water Samples

The proposed electrodes were effectively tested for the quantification of ALN in spiked water samples including distilled and tap water (Table 5), indicating good accuracy and excellent performance of the fabricated electrodes.

### 2.3. Determination of ALN in Actual Wastewater Samples from Industrial Pharmaceutical Plants

The working settings were calibrated and adjusted for the fabricated electrodes and different wastewater samples were then analyzed. The results were compared to those obtained by analyzing the samples using the official USP method [30] after sample pre-treatment by solid phase extraction (SPE) (Table 6). The performance of the fabricated electrodes for the determination of the cited drug was also validated by the application of the standard addition procedure. The recovery % was calculated for the added ALN concentrations (Table 7). The samples were analyzed directly without any prior treatment, which constitutes an excellent advantage of the suggested technique over other analytical techniques, like LC-MS/MS and ion chromatography.

### 2.4. Statistical Application

There was no significant difference between the quantification of the target drug in pure form by the fabricated electrodes and those determined by the corresponding official USP method [30]. The measured t- and F-values were found to be lower than the calculated values. The proposed method was found to be precise and exact, as the results were in good accordance with those obtained by the official method (Table 8).

## 3. Discussion

The fabricated sensors can be used to quantify ALN residues in wastewater samples without sample pre-treatment, which in turn makes the environmental monitoring task of the studied drug an easy process. To optimize the working conditions for the fabricated electrodes, the execution of ISEs was assessed by various factors, including response time, pH range, limits of linear response, the limit of detection, selectivity, and shelf life. The electrochemical performance features of the ISEs were examined as per the IUPAC standards [28]. The effect of solvent mediators was studied using three different plasticizers (DBS, NPOE, and DOP) of different polarities. NPOE as a plasticizer produced the perfect response, as the slope of its fabricated sensors were closest to the Nernstian one. This may be ascribed to the best matching of characteristics with the studied drug, which enabled the finest movement of the ions through the membranes.

The sensors displayed a fast and steady response with a low limit of detection (LOD) over a wide range of concentrations, which makes the fabricated electrode suitable for quantification and monitoring of the studied drug in wastewater samples. A constant response was attained in the pH range of 8 to 11 for the PVC and GC membrane electrodes. The response was noisy and exhibited considerable aberrations from the model Nernstian slope (30 mV/decade) besides this pH range. Regarding thermal stability of the fabricated electrodes, the LOD and slope showed insignificant variation, indicating realistic thermal stability of the fabricated membrane electrodes up to 40 °C.

The accuracy of the suggested method was checked by analyzing pure ALN samples of different concentrations using the fabricated sensors. Ruggedness and robustness are important parameters in method validation as they ensure that the proposed method is not affected by slight changes in the experimental conditions. They were checked and are shown in Table 2. Selectivity of the fabricated membranes is an important parameter in performance optimization, as it defines the scope and usefulness of any sensor in real sample measurements. Different interfering materials, including many inorganic salts with different charges, were tried. Additionally, two structurally related drugs to ALN (residronate sodium and clodronate disodium tetrahydrate) were tried as interfering materials, which was considered as a tough challenge for the fabricated electrodes’ selectivity.

The proposed electrodes were effectively tested for the quantification of ALN in spiked water samples, including distilled and tap water (Table 5). The fabricated electrodes were also applied for the quantification of the studied drug in actual wastewater samples. In order to validate the results obtained by the fabricated electrodes, the concentrations found for the actual samples were compared with those obtained by applying the official USP method [30] (Table 6), declaring good matching between the results. The standard addition technique was well applied for the actual wastewater samples, confirming the excellent performance of the fabricated electrodes (Table 7). The wastewater samples were quantified directly without any sample pre-treatment, which established the major benefit of ISEs over other analytical techniques. The monitoring of the studied drug, ALN, by the developed fabricated electrodes was found to be simple, economic, and suitable for environmental analysis.

## 4. Materials and Methods

### 4.1. Instrumentation

The measurements of the electrochemical responses were performed by Jenway’s Digital pH/mV (Model 3510, keison products, Chelmsford, UK) furnished with Ag/AgCl bi-junctional reference electrodes. A magnetic stirrer and a pH glass electrode for pH measurement were also used.

### 4.2. Chemicals and Reagents

ALN was gifted by the scientific office of Riyadh Pharma (Riyadh, Kingdom of Saudi Arabia). Its purity was evaluated as per the official USP method [30] and found to be 99.95%. Acetic acid, *o*-phosphoric acid, iron (II) ammonium sulfate, boric acid, sodium hydroxide, potassium chloride, ethanol, and tetrahydrofuran (THF) were purchased from Prolabo, France. *O*-phenanthroline, DOP, NPOE, and DBS were procured from Sigma, Germany. PVC (HMW grade) was purchased from Fluka Chemie, Germany. Carbon electrodes (C-E-02-ELEC) were purchased from American Elements (Orlando, FL, USA). Deionized water was acquired from Aquatron Automotive water still A 4000, Bibby Sterillin Ltd., (Staffordshire, UK). Ferroin (*o*-phenanthroline-iron (II) complex) was made by mixing 100 mg of *o*-phenanthroline and 20 mL of 1 × 10^−2^ M iron (II) ammonium sulfate. A clear solution of ferroin was then obtained by adding water and ethanol dropwise. Britton Robinson buffer (BRB) was prepared by stirring equivalent amounts of acetic acid (0.04 M), boric acid (0.04 M), and *o*-phosphoric acid (0.04 M). A pH range of 4 to 12 of BRB was prepared by adding 0.2 M sodium hydroxide dropwise [31].

### 4.3. Sample Collection, Preparation, and Storage

The wastewater samples were collected from an industrial pharmaceutical plant in the Riyadh region (KSA). It is located about 120 km away from King Khaled airport. The samples’ pH was adjusted to 9. Fine particulate matter from samples was filtered out through nylon membrane filters (0.45 µm). Filtrate was stored at 4 °C in the dark to circumvent any degradation of the analyte [32].

### 4.4. Standard Solutions

Standard solution of concentration 1 × 10^−1^ M was prepared by weighing, transferring, and mixing 3.25 gm of ALN in 100 mL of deionized water in a volumetric flask. Working solutions of concentrations between 1 × 10^−7^ to 1 × 10^−2^ M were made from standard stock solution by diluting with deionized water.

### 4.5. Procedures

#### 4.5.1. Precipitation of the Ion Exchanger

Five milliliters each of standard aqueous solutions of ALN (1 × 10^−1^ M) and *o*-phenanthroline-iron (II) were vortexed for about 5 min. The obtained precipitate of ALN-*o*-phenanthroline-iron (II) was processed and washed with cold deionized water till no traces of chloride were found in the AgNO_3_ test. The precipitate was dried completely and then ground to obtain a fine powder. The ALN ion exchanger was analyzed for its carbon, hydrogen, and nitrogen composition by elemental analysis.

#### 4.5.2. Sensor Fabrication

For the PVC membrane sensor, the prepared ALN ion exchanger (40 mg) was mixed with NPOE (400 mg) and PVC (170 mg) in a Petri dish of 5 cm in diameter. THF (5 mL) was added to dissolve the mixture and then the Petri dish was kept overnight at room temperature for the complete disappearance of the solvent. A membrane of thickness 0.1 mm was attained. A disk of diameter 8 mm was taken out and applied to an interchangeable PVC tip with the help of THF, which was attached at the end of the electrode’s body. Internal reference solution was made by combining the equivalent amount of ALN standard solution (1 × 10^−2^ M) and KCl (1 × 10^−2^ M). An internal reference electrode, made up of Ag/AgCl wire of diameter 1 mm, was dipped in the internal reference solution.

For the GC electrode, the membrane was prepared by immersing the top of the carbon electrode several times in ALN–NPOE–PVC solution and then kept overnight at room temperature for complete vaporization of the solvent.

#### 4.5.3. Sensor Calibration

Conditioning of the sensors was performed for 24 h by soaking them in an aqueous ALN solution (1 × 10^−2^ M). The calibration of the electrodes was carried out by immersing them in separate 100 mL beakers containing 50 mL of ALN aliquots at concentrations from 1 × 10^−7^ to 1 × 10^−2^ M, with continuous mixing in combination with Ag/AgCl reference electrodes. Between measurements, deionized water was used for washing the electrodes. The calibration curve was plotted between electrode potential and negative logarithmic concentrations of ALN and the same was utilized for the quantification of unknown samples.

#### 4.5.4. Sensor Optimization

Different factors and parameters affect the performance of the fabricated electrodes. They were studied and optimized to obtain the best electrochemical response. The electrochemical competence of the electrodes was assessed as per IUPAC recommendations [28]. To study the effect of solvent mediator type, different plasticizers of varied polarities, like DOP, DBP, and NOPE, were investigated to find optimal membrane performance. The Nernst equation was used to calculate the slope of the calibration plots. Over two months, the long-term stability and reproducibility of the electrode potentials were assessed by calculating and plotting duplicate calibration curves. The pH effects were checked by immersing the sensors in ALN standard solution of concentrations of 1 × 10^−4^ M and 1 × 10^−3^ M, over the pH range of 4 to 12 using BRB. Then, the electrode potential was plotted versus the pH of the solution. The temperature effects were also checked by immersing the sensors in ALN standard solution of concentrations ranging from 1 × 10^−7^ M to 1 × 10^−2^ M, at a temperature of 25 °C, 30 °C, and 40 °C. The execution of the proposed sensors was also established in the presence of several interfering substances using the matched potential method (MPM) and calculating the PSCs for the tested interfering substances [29]. They were calculated by carrying out a two-step experiment. In the first step, ALN standard solution 1 × 10^−3^ M was used as a reference and standard ALN was added to make the concentration 1 × 10^−2^ M, which produced a potential change of 29 mv. In the second step, the interferent was added to the same standard ALN solution 1 × 10^−3^ M to obtain the same potential change (29 mv). The selectivity coefficients were calculated as the ratio of ALN concentration to the interferent concentration which gives the same potential change in the same ALN reference solution (1 × 10^−3^ M).

### 4.6. Application

#### 4.6.1. Determination of ALN in Spiked Water Samples

Ten milliliters of ALN standard solution (1 × 10^−2^ M) was added into two 150 mL beakers each. In one beaker, 50 mL of deionized water and, in the other, 50 mL of tap water was added. The pH was adjusted to 9. The contents of each beaker were quantitatively transferred into two separate 100 mL volumetric flasks. The contents of each flask were filled to the mark using either deionized water or tap water. Fifty milliliters from each flask was transferred into two separate 150 mL beakers. The membrane sensors were immersed in concurrence with the reference electrode. The measured potentials were used to calculate ALN concentrations.

#### 4.6.2. Determination of ALN in Actual Wastewater Samples

The potentiometric measurements for 6 actual wastewater samples were taken using the proposed sensors in concurrence with the reference electrode. Calibration plots were used to obtain the unknown concentrations. The obtained results were compared to those obtained by analyzing the samples using the official USP method [30] after sample pre-treatment by SPE. Additionally, the standard addition procedure was applied by the addition of three concentration levels of pure ALN (100, 200, and 300 µg/mL) to the actual wastewater samples, then the samples (after the addition of pure ALN) were analyzed using the fabricated electrodes. The recovery % for the added amounts of pure ALN was calculated.

## 5. Conclusions

This work represents the development and application of ISEs viz. PVC membrane electrode and GC electrode, for a straightforward, simple, careful, and profound quantification of ALN in diverse samples, particularly in industrial wastewater and complex matrices, without prior treatments. The execution of ISEs was assessed by various factors, including response time, pH range, dynamic linear response ranges, the limit of detection, selectivity, and shelf life. The sensors displayed a fast and steady response with a low limit of detection (LOD) over a wide range of concentrations. A constant response was attained in the pH range of 8 to 11 for the PVC and GC membrane electrodes. The LOD and slope showed insignificant variation, indicating realistic thermal stability of the fabricated membrane electrodes up to 40 °C. Different interfering salts, including two structurally related drugs to ALN (residronate sodium and clodronate disodium tetrahydrate), were tested as interfering materials, confirming the excellent performance and selectivity of the fabricated electrodes.

The proposed method is novel in its field, as there is no published work dealing with the electrochemical quantification of ALN in environmental samples like wastewater samples. The proposed technique is superior to other analytical techniques such as LC-MS/MS and ion chromatography, as it lacks any prior sample treatment and guarantees sensitivity and suitability for environmental analysis. Additionally, the proposed method is very economic, as it does not need special and expensive instruments, unlike LC-MS/MS and ion chromatography. The sensors displayed a fast and steady response over a wide range of concentrations and pH. The fabricated sensors are found to have critical importance in monitoring environmental wastewaters containing ALN, where its occurrence may lead to adverse consequences for human health and society.

## Figures and Tables

**Table 1:** Elemental analysis of alendronate (ALN) *o*-phenanthroline–Iron (II) complex.

Parameter	Analysis %		
	C	H	N
Calculated %	56.15	4.15	11.62
Found %	55.81	4.18	11.55

**Table 2:** Electrochemical response characteristics of the fabricated electrodes.

Parameter	PVC Membrane Electrode	GC Electrode
Slope (mV decade^−1^) *	29	29
Response time (sec)	15	15
Correlation coefficient (r)	0.9995	0.9991
Working pH range	8–11	8–11
Concentration range (M)	1 × 10^−5^–1 × 10^−2^	1 × 10^−5^–1 × 10^−2^
Concentration range (µg/mL)	3.25–3250	3.25–3250
Stability (days)	25	30
Accuracy (Mean * ± SD)	100.20 ± 0.809	100.42 ± 0.929
Limit of detection (M) **	8 × 10^−6^	8 × 10^−6^
Ruggedness ^†^	103.12 * ± 2.014	102.96 * ± 1.966
Robustness ^Ψ^	100.96 * ± 0.987	99.36 * ± 0.586

* Average results of five determinations. ^†^ Comparing the results by those obtained by different sensor assemblies using Hanna digital ion analyzer. ^Ψ^ Carried out by measuring different known ALN concentrations with slight pH change (pH 9 ± 0.2). ** Lower limit of detection obtained by calculations.

**Table 3:** Profile of the potential versus –log concentration for the PVC membrane electrode and the GC electrode, at three temperatures (25 °C, 30 °C, and 40 °C) at pH 9.

−log Conc. (M)	E (mV)					
	PVC Membrane Electrode			GC Electrode		
	25 °C	30 °C	40 °C	25 °C	30 °C	40 °C
2	40	40	39.5	30	30	30
3	69	69	69	59.5	59	59
4	98	98.5	98	89	88	88
5	127	127.5	127	118	117	117
6	141	141	141	133	132	132
7	142	142	142	134	134	134

**Table 4:** Potentiometric selectivity coefficients of the proposed alendronate-selective sensors by matched potential method.

Interferent	PVC Membrane Electrode *	GC Electrode *
Magnesium chloride	5.3 × 10^−2^	5.1 × 10^−2^
Potassium sulfate	3.1 × 10^−2^	3.3 × 10^−2^
Potassium phosphate	4.1 × 10^−2^	4.5 × 10^−2^
Ammonium nitrate	7.6 × 10^−3^	7.8 × 10^−3^
Potassium carbonate	4.1 × 10^−2^	3.8 × 10^−2^
Sodium fluoride	5.2 × 10^−2^	6.1 × 10^−2^
Sodium iodide	2.7 × 10^−2^	2.9 × 10^−2^
Residronate sodium	5.5 × 10^−2^	5.6 × 10^−2^
Clodronate disodium tetrahydrate	5.9 × 10^−2^	5.8 × 10^−2^

* Average of five measurements.

**Table 5:** Determination of ALN in spiked water samples using the proposed electrodes.

Specimen	PVC Membrane Electrode	GC Electrode
Distilled water (Rec. %) *	102.57	101.89
Tap water (Rec. %) *	101.78	103.11

* Average of five measurements.

**Table 6:** Determination of ALN in actual wastewater samples from pharmaceutical plants.

Sample Number	PVC Electrode ^β^	GC Electrode ^β^	Official USP Method [30] *
Sample 1	10.33	10.99	10.63
Sample 2	15.98	15.22	15.56
Sample 3	25.76	25.52	25.65
Sample 4	32.11	32.74	32.46
Sample 5	53.25	52.45	52.84
Sample 6	78.96	77.47	78.86

**^β^** Samples are calculated in micrograms per milliliter. * USP-HPLC method using C18 column (4 µm, 25 cm × 4.6 mm, i.d.) as stationary phase, acetonitrile:methanol:0.05 M sodium citrate-dihydrogen phosphate buffer (pH 8) (25:5:70, by volumes) as mobile phase, flow rate 1.2 mL/min and UV detection at 266 nm.

**Table 7:** Application of standard addition procedure for ALN determination.

Added ALN (µg/mL)	PVC Electrode (Recovery % ± SD) *	GC Electrode (Recovery % ± SD) *
100	100.81 ± 0.831	100.79 ± 0.799
200	99.69 ± 0.731	101.55 ± 0.893
300	102.11 ± 0.921	99.49 ± 1.031

* Average of three readings.

**Table 8:** Statistical treatment of the results obtained by the suggested ion-selective electrodes in comparison with the official method.

Items	PVC Membrane Electrode	GC Electrode	The Official USP Method
Mean ± SD	100.20 ± 0.809	100.42 ± 0.929	99.81 ± 0.587
RSD	0.807	0.925	0.588
Variance	0.654	0.863	0.345
n	5	5	5
F-value (6.39) *	1.90	2.50	-
Student’s *t*-test (2.306) *	0.920	1.242	-

* Values in parentheses are the theoretical values of t and F at *p* = 0.05.

## Data Availability

The datasets used and/or analyzed during the current study are available from the corresponding author on reasonable request.

## Sample Availability

The datasets used and/or analyzed during the current study are available from the corresponding author on reasonable request.
